# How Did People with Functional Disability Experience the First COVID-19 Lockdown? A Thematic Analysis of YouTube Comments

**DOI:** 10.3390/ijerph191710550

**Published:** 2022-08-24

**Authors:** Karen A. E. Hall, Blanca Deusdad, Manuel D’Hers Del Pozo, Ángel Martínez-Hernáez

**Affiliations:** Department of Anthropology, Philosophy and Social Work, Rovira I Virgili University, 43003 Tarragona, Spain

**Keywords:** COVID-19, functional disability, disability, discrimination, isolation & social media

## Abstract

People with functional disability endure barriers to health and other services and to full participation in social life. In the context of COVID-19, this discrimination has been intensified worldwide. We examine how the experience of COVID-19 lockdown was depicted in comments to a video about functional disability and COVID published on VICE’s YouTube channel. We analysed the first 100 comments on the video, which was posted in spring 2020, during the first COVID-19 lockdown (roughly from March to June 2020, with some variations around the world). We identified four themes: lack of access to care and services, isolation and lifestyle changes, mental health consequences, and peer support. Legal regulations regarding COVID-19 and people with functional disability have not been sufficient in most countries. The COVID-19 pandemic has exposed inadequate care systems, even in Western countries with advanced social protection policies.

## 1. Introduction

The COVID-19 pandemic has highlighted the structural discrimination of people with functional disability throughout the world [1]. This scenario has been denounced by different international human rights institutions, namely the World Health Organisation, [2], European Disability Forum [3], the American Association of People with Disabilities [4,5]. These institutions highlight that vulnerable populations are most directly impacted, and the consequences are related not only to physical health, but also mental health and quality of life.

Research shows that there is a strong relationship between poverty and disability [6,7,8,9,10]. Poverty and disability aggravate each other, and this link has intensified during the pandemic, for instance, when people have lost income due to it [11,12,13], which limits the ability of this group to have sufficient resources to guarantee their subsistence during the pandemic, as well as to guarantee the necessary assistance in their daily lives.

Western countries have legal protections for people with functional disability that draw on the Convention on the Rights of Persons with Disabilities (CPWD). In 2006, CPWD spearheaded an international campaign ensuring that individuals with disabilities would play an integral part in developing national legal frameworks to protect them from discrimination, segregation and denial of services [14,15]. However, health structures to protect people with functional disability seem to have been ignored during the pandemic, giving priority to public health more broadly. In this sense, the rights of people with functional disability and their hard-won protections seemed not to apply and have a specific attention during the health crisis [16]: if it becomes possible to ignore these rights during a crisis, we must wonder whether societies could revert back to the pre-rights state in which the lives of persons with functional disability did not matter [17,18,19]. When medical professionals, governments, and policymakers face a shortage of resources, they have to decide whose needs to meet first. In this situation, “the relative value of lives” has resulted in discrimination and violence against people with functional disability [20] and how the importance of health treatment along life as a quality of life (QALY) is underestimated [16].

Lockdown, including stay-at-home orders and the closure of “non-essential” productive activities, has been one of the main strategies that states have used to quell the advance of the virus [21]. Because key services for people with functionally diversity were often considered “non-essential”, functionally disabled people faced serious difficulties in meeting their basic needs. 

Deusdad [22] and Lakhani [23] have pointed out that people with functional disability already faced serious challenges before the pandemic due to precarious health systems. For instance, they often had to move to metropolitan areas to receive adequate medical services [11]. There has been some coverage in the press of the situation of people living with functional disability during the pandemic [24], but little academic research has emerged so far [18,25]. The existing research denounces the hurdles people with functional disability faced when mobility restrictions blocked their access to essential therapies for months. In Western countries such as Spain, Canada, and Italy the needs of people with disabilities and older adults were not taken into account [22,26]. To the contrary, they became invisible and their need for physical therapy, follow-up treatment, periodic medical consultations, and leisure activities was considered non-priority [27].

Despite the fact that functional disability does not in itself increase the risk of contracting COVID-19, several factors—such as community-based living and the need for close contact with care providers—increased the vulnerability of this population [28]. Therefore, it is critical to reflect on the lockdown experiences of people living with functional disability. 

Research about disability in the context of COVID-19 is beginning to emerge. Some researchers have focused on the policies that governments need to implement to guarantee the right of people with functional disability to efficient healthcare [28,29,30,31,32]. Others have analysed the consequences of COVID-19 for people with intellectual disabilities [33,34]. Finally, some researchers have described the psychological effects of COVID-19 on people with functional disability [35,36]. To our knowledge, no published research has yet addressed how the experiences of COVID-19 lockdown of people living with disabilities have been depicted in public discourse. Moreover, very little information is available about how people with functional disability lived through the first lockdown, when research efforts around the world were severely constrained. We focus on how people with functional disability reported their experiences of COVID-19 lockdown in a sample of YouTube comments.

## 2. Methods

We conducted a qualitative thematic analysis of comments about the experience of lockdown of people with functional disability, as expressed in their comments about a video published by VICE on YouTube. (https://www.youtube.com/watch?v=-aGUuA5aDic&list=PLJZQF4tvTgMR3Yq9g6WfJb4IoKZpe-lpH, accessed on 17 August 2022). In choosing this source of data, we follow Way [37], who offers an analysis of YouTube videos to show how marginalised groups can use forms of expression such as music or metaphors to denounce their situation explicitly or implicitly [37]. We chose thematic analysis because it allows us to detect similarities and differences between participants [38], particularly situations experienced by people with functional disability, during the first COVID-19 pandemic lockdown, even in Western countries. This method is also a more direct and powerful way to grasp the statements of people with functional disability during the pandemic. At the same time this approach requires involvement and interpretation by researchers [39]. Since one of the authors is a person with functional disability, this method was considered the most suitable.

The VICE video contained interviews with three people with functional disability (physical disabilities). The interviewer asked them about their personal experience during the first stay-at-home order:

1. How has your life changed because of COVID-19?
2. Has the COVID-19 outbreak prevented you from receiving care required to manage your disability?
3. What social justice issues have the social distancing policies raised for disabled people?
4. What does disability justice look like within the COVID-19 outbreak and beyond?

These questions led not only to the interviewees’ responses but to those of the commenters. The interviewees talked needing hands-on care and not being able to maintain social distance from their caregivers. They reported feeling excluded from the planning of how to meet the needs of people with functional disability during the pandemic. 

Researchers have used YouTube and other social media sites to take advantage of publicly available data, this is to say, open-source data, which has been anonymized. Another advantage is that such sites provide a platform for powerless people to describe their experiences and express their concerns [40] and to talk about discrimination and lack of social justice [37]. In terms of the reliability of YouTube comments, Nguyen and Allen [41] found that the comments they examined in a patient feedback study were an accurate reflection of participants’ medical histories. As several authors have pointed out, social media gives ordinary people the opportunity to voice their experiences and needs [40,42,43].

### 2.1. Sample

The VICE video appeared on YouTube in April 2020 and at the time of writing, it has more than 600 comments and 700,000 views. We analysed the three interviewees’ statements from the video and extracted the first 100 comments in which the commenter reported having functional disability and described his or her experiences during lockdown. Most comments ranged in length from one to two paragraphs. We used these comments as a way to access the fleeting experiences of people during the first lockdown, when the global health crisis meant that few researchers were able to conduct qualitative interviews about the unfolding disaster. Although the source was open-source data, the commentors’ names have been anonymised for the purpose of this paper. 

### 2.2. Coding and Themes

To analyse the comments, we used thematic analysis, which is an inductive, qualitative method that permits a fresh approach to the data [39,44,45]. This approach allowed us to note similarities, differences, and trends across commenters’ reported experiences. We constructed a set of 19 codes inductively after reading the data, without working from a pre-conceived set of categories [46]. We then grouped the codes into four themes (Table 1). This way of analysing data promotes reflexive thinking [47,48,49].

Following Ramalho, Adams, Huggard & Hoare [49], we point out the epistemological background of the researchers involved in this study. Our backgrounds are in anthropology and social work and all four of us are based in Europe. One author has functional disability and two come from developing countries, where protections for people with functional disability are less thoroughly codified than in Europe. We have drawn on our diverse personal backgrounds in interpreting the data.

## 3. Findings

We identified four key themes in commenters’ reports about their experiences of COVID-19 lockdown: lack of access to care and services, isolation and lifestyle changes, mental health consequences, and peer support. We created these themes by grouping the initial set of 19 codes into bigger umbrella categories (Table 1). 

### 3.1. Lack of Access to Care and Services

The three interviewees from the video denounced ways of ableism how the people were treated during the pandemic. For instance, in hospitals: people do not listen to disabled people in the hospital (Interviewee 1). Care services for doing the activities of daily life were reduced; for instance, instead of having a shower per day only they had it every two or three days, decreasing their quality of life. Numerous commenters on the video reported also that during the lockdown, health services had been postponed or directly cancelled. For example, commenters reported that the services they depended on were deemed “non-essential”:

*I am disabled due to chronic pain. I depend on steroid injections in my spine to remain mobile and help manage my pain. My pain management doctor won’t see people in the office and injections are seen as a non-essential procedure. I’m terrified of how much pain I’m going to have and how debilitated I’m going to be once normal society resumes*.

(Jon, comment 2)

One commenter mentioned that lack of access to regular care for depression was particularly difficult:

*Can’t see my doctors, they only take emergencies, and telemedicine is wholly inadequate to even address my physical health and my depression is crushing me from the inside out*.

(Anthony, comment 75)

Another commenter mentioned the difficulty of accessing controlled medications:

*My aunt is disabled and in a wheelchair with excoriating bone on bone pain that requires controlled medications. She has no family doctor. The clinics are closed and it’s very difficult for her to come in person if they are open. She tried calling the telemedicine doctors, but they cannot prescribe her meds unless she is seen in person*.

(Sam, comment 72)

Others mentioned the importance of hands-on treatment such as physical therapy and chiropractic:

*Most of my appointments are completely cancelled, sometimes at short notice, sometimes they’re phone consultations (which freak me out) but now I can’t have my x-rays or specialist appointments. Physio is reduced to every 2 weeks, therapy is basically just a 30 min catch up rather than structured work*.

(Greta, comment 92)

*I depend on chiropractic treatment and massage therapy to function well. Luckily, I’m prepared with pain medication as a backup, plus I’m only working 50% of what I normally work*.

(James, comment 45)

These examples show the suffering caused to people with functional disability when medical services they needed were classified as “non-essential.” Commenters also reported discomfort when the lack of care services made them vulnerable and/or forced them to rely on family members. Some people with functional limitations were left unattended in their homes. Some caregivers visited their clients with functional disability less frequently due to a fear of contagion, leaving commenters without the necessary support:

*I have a paid caregiver. She now refuses to do many of my ADLs including laundry, shopping, cooking, and other care. She’s supposed to come 4 days a week and is only coming 2 or 3. It’s difficult to find a new caregiver in the best of times; it’s nearly impossible in this pandemic.*

(Julie, comment 27)

An outcome of the pandemic is that the leisure programmes for persons with disabilities participated in were cancelled, so many were forced to rely on their families to fill the gap:

*How COVID is really affecting me is I can’t go to my day program because of this virus🦠 it really upset me that this virus is preventing me from going out in my community and can’t see my friends just hate being around my family now been around them too long yes I love them but I need some me time*.

(Anne, comment 40)

Similarly, the unavailability of social supports during lockdown led people with functional disability to depend on others, which some described as an emotional and financial burden:

*When you are disabled and depend on other people this creates many hardships. It’s very exhausting, expensive and horrific. Many support systems are not available*.

(Marc, comment 26)

### 3.2. Isolation and Lifestyle Changes

The three interviewees from the video and numerous commenters of it referred to loneliness, pointing out that isolation is nothing new for many people with functional disability:

A lot of abled people are freaking out about quarantine and disabled people are not (Interview 3).

*Carona is barely changing my lifestyle*.

(Peter, comment 36)

*I’m disabled, but I haven’t talked to a real-life person in almost two years, so I’m exactly the same. I wouldn’t even know Coronavirus was happening if it wasn’t all over YouTube*.

(Alan, comment 13)

Yes, physically disabled, and also mentally disabled. I have had severe depression since diagnosed over 50 years ago. Before the onset of this pandemic, my routine already consisted of isolating most of the time and barely being in touch with anyone. Basically, the only difference is the mask.

*I’m required to wear in Sonoma County CA*.

(Vic, commentor, 92)

*Something making you unable to sit used to being depressed coronavirus is barely changing my lifestyle short of not working*.

(Peter, comment 67)

Sometimes the similarity of life before and during COVID for people with functional disability is stated more implicitly. The following commenter compares the feeling of isolation caused by lockdown to the feeling of isolation he or she already felt as a disabled person without family:

*I’m disabled as well and I have no family so I completely understand the feeling of isolation*.

(Kamil, comment 38)

We argue not that lockdown was not a burden for people with functional disability, but rather that the similarity to their pre-pandemic life points out important ways in which they were already systematically excluded from social life.

In some cases, the isolation of the pandemic did cause new hardships for people with functional disability, especially for people with depression. Social isolation is specifically contraindicated for people with severe depression, meaning that the COVID-19 lockdown was a grave threat to their health and wellbeing [16] as the following comment illustrates:

*I have severe depression and gender dysphoria. Keeping me in the house is not good for me. They need to make haircuts and clothing stores necessities*.

(Silvie, comment 43)

This commenter points out that removing access to seemingly superfluous services could cause severe suffering for people with depression.

### 3.3. Mental Health Consequences 

There was little health equity at the biggening of the COVID-19 outbreak in 2020. Many countries went into total lockdown [50], and extreme isolation became a mental health stressor [51]. These ‘mental health stresses’ can be seen in multiple situations that people with functional disability faced are which are unknown and invisible for the rest of the population. Persons with disabilities not only had to protect themselves but also faced additional contagion prevention measures, such as the need to have an outdoor wheelchair and an indoor wheelchair (or to clean the wheels of their wheelchair before entering their home after going outside). 

One consequence that persons with functional disability are enduring during the pandemic period is that necessary health services have been cancelled or postponed. For example, those with chronic pain have no access to their medication and fear that their health will deteriorate.

This situation has had consequences on mental health and wellbeing. People’s routines were disrupted and they felt unsafe, uncertain, and vulnerable. People were under pressure, with symptoms of depression and anxiety and sleeping difficulties, leading to psychological risk, as revealed in the following comments:

*I am disabled due to chronic pain. I depend on steroid injections in my spine to remain mobile and help manage my pain. My pain management doctor won’t see people in the office and injections are seen as a non-essential procedure. I’m terrified of how much pain I’m going to have and how debilitated I’m going to be once normal society resumes*.

(Jon, comment 3)

Some states in the US, in particular, cancelled services they deemed “nonessential” or elective treatment, this is to say services that did not imply a disease treatment. However, for many persons with functional disability these are necessary services:

*I depend on chiropractic treatment and massage therapy to function well. Luckily I’m prepared with pain medication as a backup, plus I’m only working 50% of what I normally work*.

(James, comment 45)

### 3.4. Peer Support

One positive element that we detected in the comments is the mutual support that people with functional disability offered each other. 

In the following example the commenter encourages others to “trust” him or her and believe that they can find support in their community:

*Normally we have to stay inside on a daily basis because the world does not accept us. COVID have hit me close to home. I have autism and making friends has always been a struggle to me. Luckily, I did find amazing people who accepted me for who I am eventually, it might not seem like it, but there are people out there who truly cares and who will treat you like anybody else despite your disability, unfortunately those people are very difficult to find, but they do exist, trust me!*

(Steven, comment 12)

Others used this comments section to reach out explicitly to other commenters and even the makers of the video:

*At Emma Rose I have Twitter if you ever would like to talk over there, I am sure we can sorry we don’t live close to each other but we could still be online friends if you want*.

(Alan, comment 16)

*Thank you for making this video guys and thank you for all the wonderful people speaking out for us disabled housebound people we matter love you all*.

(Martha, comment 35)

## 4. Discussion

We have focused on the experiences of people with functional disability during the first wave of the COVID-19 pandemic, as expressed in the comments section of a video posted on YouTube. We identified four themes: a lack of access to care and services, isolation and lifestyle changes, mental health consequences and peer support. 

First, in terms of the lack of access to care and services, commenters faced the unwillingness of care workers to provide essential health services because they were afraid of contracting COVID-19. The comments also reflect the lack of priority given to the needs of people with functional disability in planning the COVID-19 response [27]. In particular, rehabilitation systems and other supportive measures were not been a priority [11], with dire consequences for people with functional disability. The stigma surrounding disability makes it difficult for people with functional disability to participate in society and in the public health decision processes, which have been a particular focus during the COVID-19 health crisis. Forms of ableism [52,53] were denounced in the VICE video and in the selected comments.

Second, isolation and lifestyle changes have been a major issue during the pandemic. Brooke and Jackson [54] have noted the consequences for people with functional disability of the social isolation resulting from confinement. Some authors conclude that people with functional disability were more likely to report loneliness during COVID-19 lockdown, particularly younger people who were living with little social and economic support and couples living in neighborhoods deprivation and in retirement-age households [55,56]. In contrast, in our study, several commenters pointed out that the lockdown had little impact on their social isolation, which was already severe. This is a key point for policymakers as they consider how to support the quality of life of people with functional disability both in pandemic times and “normal” ones.

Third, mental health for people with functional disability has worsened during the pandemic. The pandemic generated and/or exacerbated several stressors for mental health, which are affecting more people with functional disability disproportionately, such as feeling isolated and experiencing difficulties in accessing care services [51,57]. Basic services for people with functional disability were cancelled because they were not considered essential. These decisions have had negative consequences for the wellbeing of people with functional disability and their mental health conditions. Ableism became rampant, and there is still a need for a discourse around the fact that ‘disabled lives matters’ [58,59].

Fourth, we noted that commenters used the comments section to provide support to peers and create a sense of community. This finding points to the fact that fostering online communication has been a silver lining of the pandemic. Considerable previous research about social media has highlighted its negative effects, for instance, on learning [60,61], although some positive effects have also been found [62,63]. Research during lockdown points both to possible positive effects and the dangers of fake news) [64]. Despite widespread concern about toxic social media cultures, we have suggested that social media sites such as YouTube can be a tool for people with functional disability to enter the public sphere, describe their experiences, express their emotions, and offer each other support. 

### Limitations

Boyle et al. [28] have noted the difficulty in collecting data about how people with functional disabilities have experienced the pandemic. We have taken advantage of posted social media comments to gain access to lived experience at the heigh of the first lockdown, when research around the world was severely limited. This approach has some drawbacks, since we are not able to contact commenters, verify the veracity of the comments, or track the relationship between comments and sociodemographic variables, such as gender, place of residence and socioeconomic status. Furthermore, commenters respond to interviewees in the VICE video, and consequently, their opinions are influenced and adapted to them. However, in addition to providing us with data from a time when little data was available, the comments have the advantage of anonymity, meaning that commenters had little incentive to misrepresent themselves and their experiences. To complement our findings, further qualitative research could be conducted, using in-depth interviews and focus groups to ask people with functional disability to recall their experiences of lockdown and to add the gender perspective.

## 5. Conclusions

People with functional disability were disproportionately affected by the COVID-19 lockdown, because they depend on regular medical services and care support. Many people with functional disability were isolated before the pandemic, and the pandemic worsened their situation. This health crisis has revealed the insufficiency of care systems, even in Western societies, and has increased the neglect and discrimination suffered by people with functional disability. The disability community found that COVID-19 regulations served as a barrier to their meagre rights and equity in the obvious deregulations of therapies, doctor visits and access to medications.

Lockdown has highlighted the practical advantages of extracting data from social media. We have shown that the analysis of social media can be an effective way to learn about the experiences of people with functional disability, especially in times of limited mobility. Examining social media posts from the height of the initial lockdown in spring 2020 allows us to capture their feelings of abandonment and loneliness while they were literally stuck at home. Further research on the experiences of people with functional disability can help inform a broader investigation into the neglect of human rights during the pandemic and policy interventions to ensure that the needs of all people are met in future global health crises.

## Figures and Tables

**Table 1:** Themes and Codes.

Themes	Codes	No. of Comments
1. Lack of access to care and services	Economic consequences	49
	Segregation	
	Inequities of the healthcare system in its bias against the disabled	
	Loss of home care services	
	Discrimination increased during pandemic	
	Ableism	
2. Isolation and lifestyle changes	Isolation	17
	Increased negation ^1^	
	The mismanagement of COVID-19 leads to discrimination	
	Social programs cancelled	
	Lost income	
	No changes in their lives	
3. Mental health consequences	Anxiety and fear of death	10
	Depression	
	Isolation and rejection	
4. Peer support	Support from VICE	30
	‘our voices matter’	
	Community support	
	Self sufficiency	
	Digital peer support	

^1^ Referring to two negatives impacting each other and rising the effect of an already difficult situation e.g., disability and COVID-19.

## Data Availability

The data used in this study is available at VICE video on YouTube: https://www.youtube.com/watch?v=-aGUuA5aDic&list=PLJZQF4tvTgMR3Yq9g6WfJb4IoKZpe-lpH (accessed on 17 August 2022).
